# Social and environmental risk factors for the accidental drowning of children under five in China

**DOI:** 10.1186/s12889-020-09650-0

**Published:** 2020-10-15

**Authors:** Meixian Wang, Yuxi Liu, Leni Kang, Chunhua He, Lei Miao, Jianwen Huang, Xiaoyan He, Jun Zhu, Juan Liang, Qi Li, Yanping Wang, Hanmin Liu

**Affiliations:** 1 Key Laboratory of Birth Defects and Related Diseases of Women and Children (Sichuan University), Ministry of Education, West China Second University Hospital, Chengdu, China; 2 National Office for Maternal and Child Health Surveillance of China, West China Second University Hospital, Sichuan University, Chengdu, Sichuan China; 3 Meishan Maternal and Child Health Care Hospital, Meishan, Sichuan China; 4 Chengdu Qingyang Maternal and Child Health and Family Planning Service Center, Chengdu, Sichuan China; 5 Department of Pediatrics, West China Second University Hospital, Sichuan University, Chengdu, China

**Keywords:** Accidental drowning, Risk factors, Children, China

## Abstract

**Background:**

Accidental drowning of children under five is a serious problem in China. The present study analyzed data on environmental and sociodemographic factors and on primary caregivers of drowned children to understand factors that may contribute to this problem.

**Methods:**

The present study collected information on 563 cases of drowning in children under five from October 1, 2015, to September 30, 2016, in 334 sampling districts in China. Primary caregivers were interviewed individually using the Drowning Mortality among Children under 5 Questionnaire.

**Results:**

Most drowned children under 5 years old were boys, and 71.6% lived within 100 m of a body of water. The drownings primarily occurred in ponds, canals, rivers, and wells, and over 90% of these water bodies had no safety measures. There were 28.1% of primary caregivers who did not provide full-time care for the children, and 83.1% of them had no knowledge of first aid skills for drowning.

**Conclusion:**

Encouraging kindergarten enrollment and providing safety education for children may reduce drowning in children under 5 years of age. Public water body protection measures should be strengthened to prevent children from drowning. Encouraging primary caregivers to care full-time for the children and learning first aid skills for drowning may also help reduce fatalities.

## Background

Drowning is a serious contributor to mortality and disability. With nearly 372,000 deaths reportedly due to drowning each year worldwide, it is a leading cause of child mortality [1]. Drowning that does not end in death may lead to cognitive difficulties, hypoxic-ischemic brain injury, cardiac abnormalities, and adult respiratory disease syndrome later in life [2–4]. Although the mortality rate due to drowning is decreasing [5], the associated economic burden remains significant. For example, the combined effects of fatal and nonfatal drowning in 2017–2018 cost 1.47 billion AUD (over 7.1 billion Chinese renminbi) [6].

Drowning is the main cause of accidental deaths in children worldwide under 5 years of age [7]. As the world’s largest developing country, China has a large population and abundant water bodies in most regions, and child drowning is also a major problem in China. In response, China has made many efforts to reduce the accidental drowning mortality rate in children, and relevant health education is actively performed at the national level. The National Health Commission of the People’s Republic of China released the “Chinese citizens’ health literacy: basic knowledge and skills guidelines in 2015”, which called for the strengthening of child supervision and education to prevent children from approaching dangerous waters [8]. Some regional governments issued a number of safety documents, including “Notice on the prevention of drowning in adolescents and children in 2019” and “Notice on doing a good job in preventing drowning of adolescents and children in 2019” [9, 10]. The Education Bureaus of many provinces also issued notices on the prevention of drowning, such as the “Notification of education on swimming safety for primary and middle school students” and the “Prohibition of bathing in rivers during summer vacation” [11].

Despite these and other preventative efforts, drowning remains an important cause of unintentional death in Chinese children [12–17]. Because of the absence of national-level data, we performed individual interviews with primary caregivers of drowned children from various parts of the country (registered in the National Maternal and Child Health Surveillance System) to elucidate the characteristics and risk factors of drowning deaths in children under five. The surveillance districts were evenly distributed in 31 provinces (autonomous regions and municipalities) of China to ensure representation of the entire country and different regions. Our results may help improve preventive measures and provide recommendations to reduce drowning mortality in this vulnerable age group.

## Methods

### Study subjects

The survey covered all 334 districts in the National Maternal and Child Health Surveillance System (NMCHSS) and 31 provinces, autonomous regions, and municipalities of China. The surveillance subject included all children under 5 in local resident households, adoptive children, and children in nonlocal resident households whose mothers lived in the surveillance districts for more than 1 year. The NMCHSS helps the national and local provincial health management departments supervise and guide health surveillance. Further details about the NMCHSS are described elsewhere [18]. The survey time period was October 1, 2015, to September 30, 2016, and there were 563 of children under 5 years in surveillance districts who died from drowning and are included in the present survey. The research team participated in the research design, questionnaire design, and data collection. We were also responsible for descriptive analysis and reporting.

### Questionnaire

Collected data were in the form of responses of primary caregivers or other family members to the Drowning Mortality among Children under 5 Questionnaire, which was designed by the Chinese National Health Commission and UNICEF to gain information on children under five who died due to drowning. The questionnaire contains four parts: basic information about children, caregivers and families, and the circumstances of the drowning. All respondents provided informed consent for their anonymized responses to be analyzed and published.

### Data collection and quality control

A trained investigator in each district branch of the National Maternal and Child Health Surveillance System was responsible for the implementation and conduct of the survey, as well as for interviewee surveying, questionnaire completion, quality control of the data, and reporting of the results. The interviews with respondents were performed within 3 months after the child’s death. At the beginning of the interview, the investigator read the questionnaire description and corresponding notes to the respondent and was allowed to call the person in charge at any time if he or she had any questions. The same investigator checked the completeness and reliability of the data after the interview. The completed questionnaires were submitted stepwise at the district, county, prefecture, city, and provincial levels of maternal and child health care centers to the National Office of Maternal and Child Health Surveillance.

### Statistical analysis

EpiData (version 3.1, The EpiData Association, Odense, Denmark) was used to establish the database. The questionnaires were double entered with logic and consistency checks. Proportions were calculated to describe the main results. Data were analyzed using SPSS 22.0 (IBM, Armonk, NY, USA).

## Results

### Sociodemographic variables associated with children under five who drowned in 334 districts in China

#### Descriptive characteristics of drowned children

**Fig. 1:**
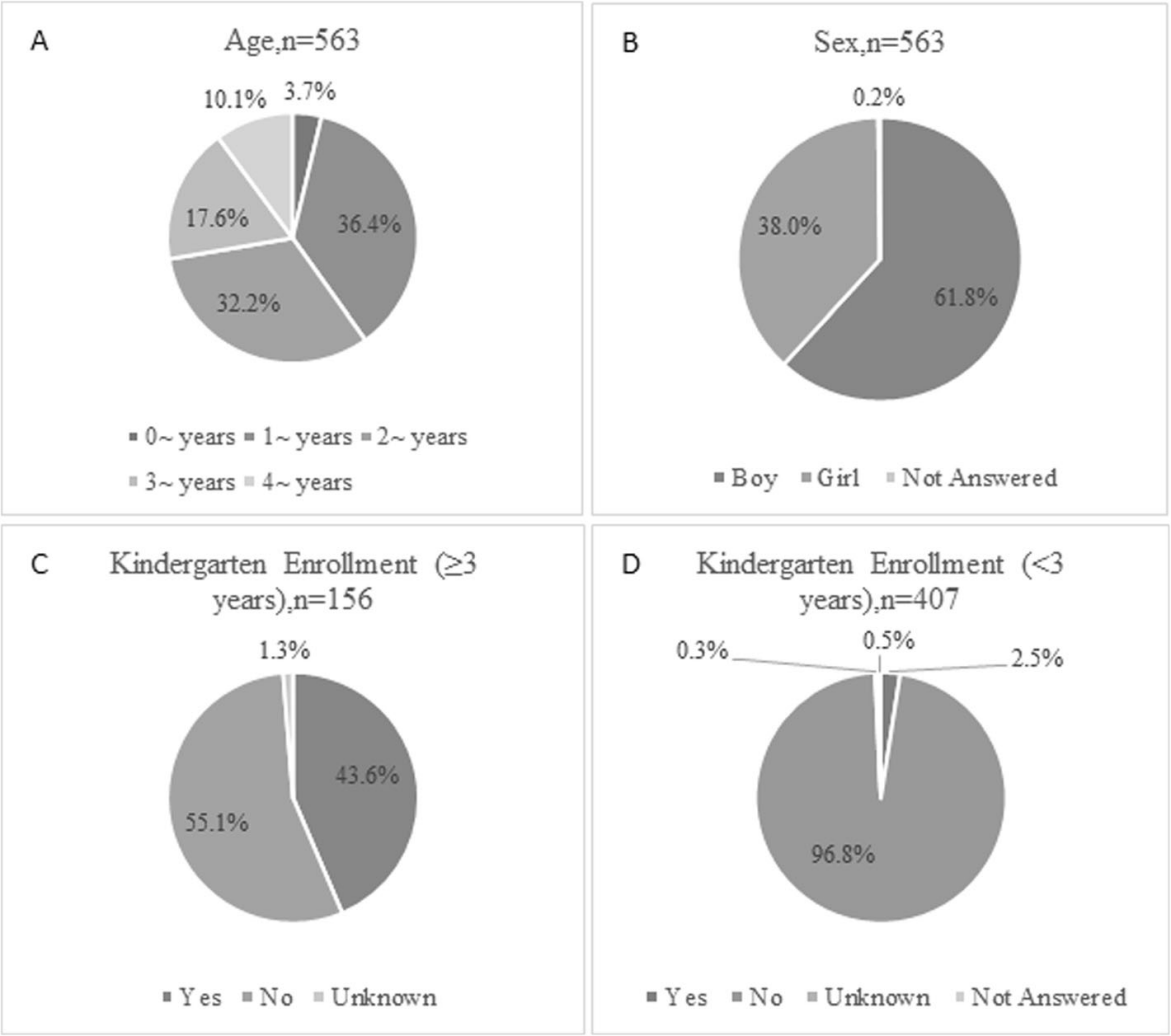
The descriptive characteristics of drowned children

The investigation included 563 children under 5 years who drowned in 334 counties in China. The proportion of male-to-female children who drowned was approximately 3:2 (348:214). Among these children, the 1 ~ and 2 ~ year age group children each represented over 30%, and the 3 ~ year age group children were the next highest group at 17.6%. Although 3 years was the age required to enroll in kindergarten, 55.2% of drowned children aged 3 years and over were not enrolled in kindergarten (See Fig. 1).

#### Safety education and risky drowning behaviors of children

The investigation also collected the safety education status of children 1 year and older. The safety education included knowing places of frequent drowning, avoiding risky drowning behaviors, understanding how to save themselves after submersion in water, and understanding how to seek help when others drowning. However, only 20.7% of children received safety education. Since playing near bodies of water may increase the risk of drowning, the investigation also surveyed risky drowning behaviors in all children. The results suggested that 3.6% of children always played near bodies of water, and 55.2% of children sometimes played near bodies of water. Moreover, 75.7% of children were playing near the body of water at the time of drowning, and 4.6% of children were playing in the body of water (See Table 1).

**Table 1:** Safety education status and activities of children under five in China

	N(%)
**Accidental injury safety education (≥1 years),** ***N*** **= 542**	
Yes	112 (20.7)
No	384 (70.9)
Unknown	23 (4.2)
Not Answered	23 (4.2)
**Playing near water body,** ***N*** **= 563**	
Always	20 (3.6)
Sometimes	311 (55.2)
Never	206 (36.6)
Unknown	21 (3.7)
Not Answered	5 (0.9)
**Activities of children under 5 years old at the time of drowning, N = 563**	
Playing near water body	426 (75.7)
Playing in water body	26 (4.6)
Slip during wading	11 (2.0)
Playing on the frozen river/lake	3 (0.5)
On the way to and from kindergarten	2 (0.4)
Bathing	2 (0.4)
Swimming	1 (0.2)
Other	43 (7.6)
Not Answered	9 (1.6)
Refused to Answer	8 (1.4)
Unknown	32 (5.7)

### Characteristics of caregivers of drowned children under five in China

#### Sociodemographics of primary caregivers

We examined the primary caregivers for each of the 563 drowned children and found that most of them (70.0%) were aged between 20 and 40 years old, and 21.0% were over 50 years old. Mothers composed a significant proportion of primary caregivers (70.7%), and approximately one-quarter (24.2%) of primary caregivers were grandparents. Only 2.0% of caregivers held some form of a college degree. The vast majority (90.2%) attained a junior high school education or less. The definition of drowning first aid skills in this investigation was that the primary caregivers could notice signs of drowning and complete first aid for drowning, which included the removal of foreign bodies in the respiratory tract, helping to extract fluid, and cardiopulmonary resuscitation. Drowning first aid skills were mostly lacking, and 83.1% of primary caregivers reported having received no training. ^*^

#### Activities of primary caregivers at the time of drowning

**Fig. 2:**
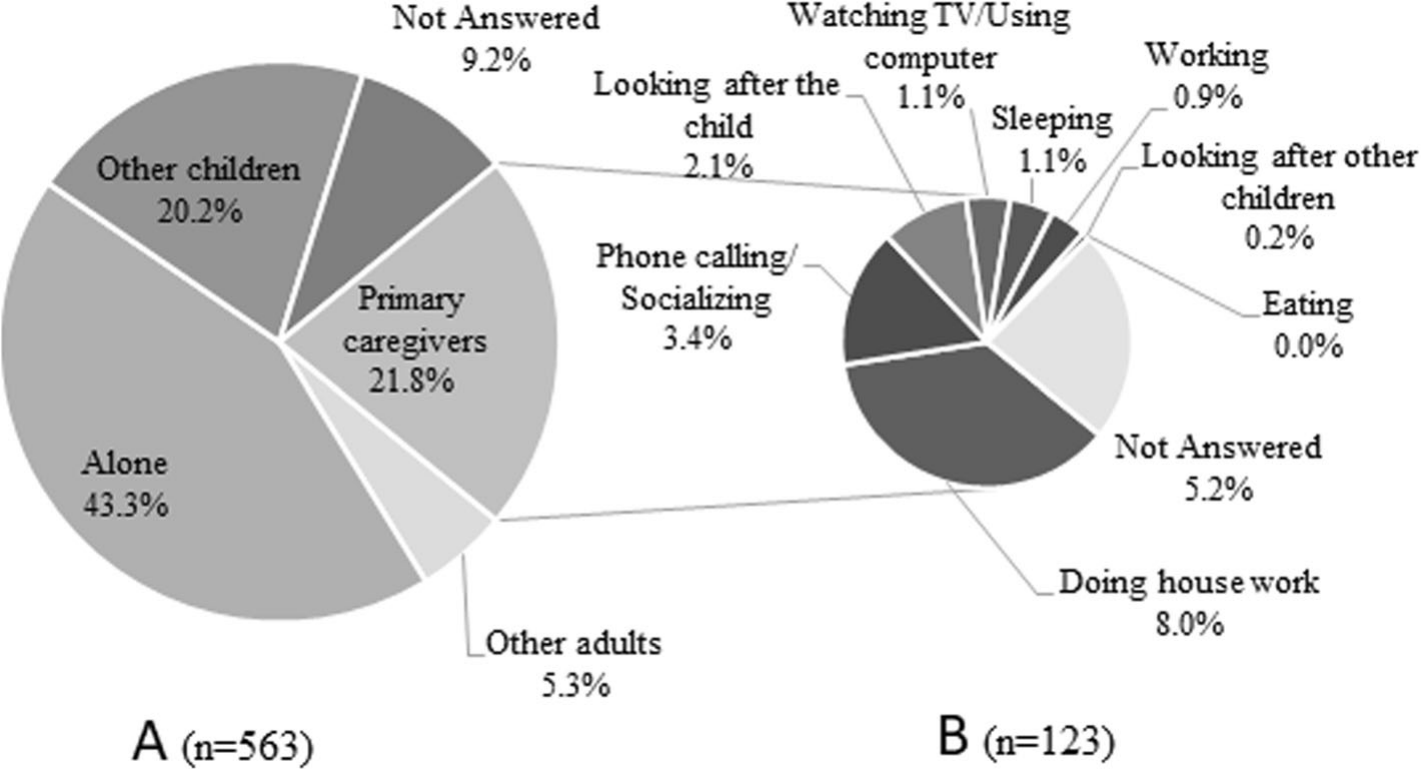
Caregiver presence and engagement at the time of drowning of Chinese children under 5 years old. A) Who was present during the child’s drowning. B) The behavior of the primary caregiver at the time of the child’s drowning

A total of 28.1% of primary caregivers reported failing to provide full-time (which was defined as the caregiver having no permanent job and only looking after one child) care to the child, which may help explain why 43.3% of children were alone (which was defined as the child being completely unsupervised) at the time of drowning (See Fig. 2). Smaller proportions of drowned children were accompanied by a primary caregiver (21.9%) or other children (20.3%). Of the primary caregivers who were with the child at the time of drowning, only 12.8% were looking after the child; 47.9% were doing housework, and 20.2% were using the phone or socializing.

### Environmental characteristics of children under 5 who drowned in 334 districts in China

#### Locations and types of bodies of water

Ponds, canals, rivers, and wells accounted for the most drowning locations (80.8%), followed by home (12.1%) (See Table 2). The results showed age differences between the drowning body of water with increasing age. The proportion of bathtub and water tank/bucket drownings decreased with age, and the proportion of pond, canal, river, and well drownings significantly increased with age.

**Table 2:** Location and type of water body involved in drowning of children under 5 years old in 334 districts in China, n(%)

Variable	Age, year					Total
	0~	1~	2~	3~	4~	
**Location of drowning**						
Ponds, canals, rivers, wells	10 (47.6)	164 (80.0)	148 (81.8)	83 (83.8)	50 (87.7)	455 (80.8)
Home	9 (42.9)	29 (14.2)	14 (7.7)	13 (13.1)	3 (5.3)	68 (12.1)
Farm (excluding home)	0 (0.0)	3 (1.5)	5 (2.8)	1 (1.0)	1 (1.8)	10 (1.8)
Factory or construction site	1 (4.8)	0 (0.0)	2 (1.1)	1 (1.0)	0 (0.0)	4 (0.7)
School (including kindergarten)	0 (0.0)	0 (0.0)	1 (0.6)	0 (0.0)	0 (0.0)	1 (0.2)
Sport place	0 (0.0)	0 (0.0)	0 (0.0)	0 (0.0)	1 (1.8)	1 (0.2)
Traffic place (such as station/ferry/pier)	0 (0.0)	0 (0.0)	1 (0.6)	0 (0.0)	0 (0.0)	1 (0.2)
Other	0 (0.0)	7 (3.4)	9 (5.0)	0 (0.0)	2 (3.5)	18 (3.2)
Not Answered	1 (4.8)	1 (0.5)	0 (0.0)	1 (1.0)	0 (0.0)	3 (0.5)
Unknown	0 (0.0)	1 (0.5)	1 (0.6)	0 (0.0)	0 (0.0)	2 (0.4)
**Total**	21 (100.0)	205 (100.0)	181 (100.0)	99 (100.0)	57 (100.0)	563 (100.0)
**Type of water body**						
Ditch	6 (28.6)	66 (32.2)	44 (24.3)	31 (31.3)	11 (19.3)	158 (28.1)
Pool (fishpond)	4 (19.1)	56 (27.3)	42 (23.2)	25 (25.3)	15 (26.3)	142 (25.2)
River /lake/sea	2 (9.5)	12 (5.9)	34 (18.8)	18 (18.2)	18 (31.6)	84 (14.9)
Reservoir	0 (0.0)	15 (7.3)	18 (9.9)	9 (9.1)	7 (12.3)	49 (8.7)
Well	0 (0.0)	6 (2.9)	13 (7.2)	5 (5.1)	0 (0.0)	24 (4.3)
Water tank/bucket	3 (14.3)	13 (6.3)	2 (1.1)	0 (0.0)	0 (0.0)	18 (3.2)
Open cesspool	2 (9.5)	4 (2.0)	5 (2.8)	3 (3.0)	2 (3.5)	16 (2.8)
Bathtub	1 (4.8)	4 (2.0)	1 (0.6)	0 (0.0)	0 (0.0)	6 (1.1)
Swimming pool	0 (0.0)	0 (0.0)	0 (0.0)	0 (0.0)	1 (1.8)	1 (0.2)
Other (such as washing machine and boilers)	1 (4.8)	21 (10.2)	21 (11.6)	7 (7.1)	3 (5.3)	53 (9.4)
Not Answered	2 (9.5)	7 (3.4)	1 (0.6)	0 (0.0)	0 (0.0)	10 (1.8)
Unknown	0 (0.0)	1 (0.5)	0 (0.0)	1 (1.0)	0 (0.0)	2 (0.4)
**Total**	21 (100.0)	205 (100.0)	181 (100.0)	99 (100.0)	57 (100.0)	563 (100.0)

A higher percentage of older children drowned away from home (87.7% of 4 ~ year age group children vs. 47.6% of 0 ~ year age group children), and younger children were more likely to drown at home (42.9% of 0 ~ year age group children vs. 5.3% of 4 ~ year age group children) (See Table 2).

#### Environmental risk factors for drowning near children’s residences

**Fig. 3:**
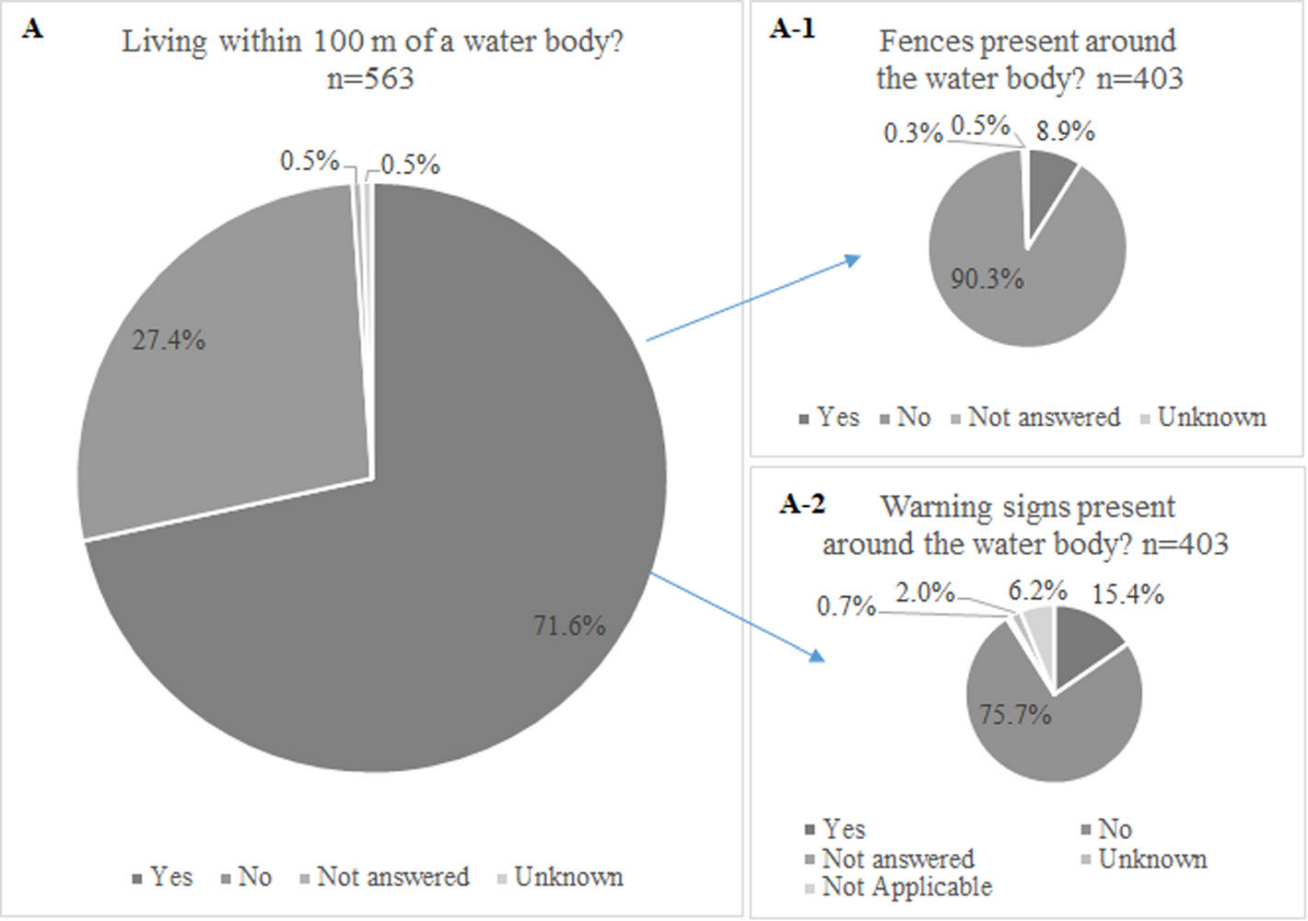
Characteristics of bodies of water within 100 m of the homes of drowned children under 5 years in China

The survey indicated that 403 drowned children (71.6%) lived within 100 m of a body of water (See Fig. 3). Approximately 90% of these bodies of water were openly accessible without any fencing. For public bodies of water, 80.7% lacked any warning signs. For bodies of water located further than 100 m from the children’s homes, only 8 of 563 could be completely covered to provide protection (See Table 3). Of the 62 bodies of water that had warning signs, the signs were difficult to see at more than half of the sites.

**Table 3:** Safety precautions for bodies of water in China where a child under 5 years old drowned

Safety precaution	N(%)
**Presence of fence (except river /lake/sea),** ***N*** **= 479**	
Yes	23 (4.8)
No	442 (92.3)
Not Answered	7 (1.5)
Unknown	7 (1.5)
**Covered, N = 563**	
Uncovered	314 (55.8)
Cannot be covered	210 (37.3)
Covered partly	18 (3.2)
Completely covered	8 (1.4)
Other	1 (0.2)
Not Answered	10 (1.8)
Unknown	2 (0.4)
**Warning signs (public bodies of water), N = 563**	
Yes, the signs are easy to notice	28 (5.0)
Yes, the signs are not easy to notice	34 (6.0)
No	465 (82.6)
Not Answered	13 (2.3)
Unknown	23 (4.1)

#### Environmental risk factors for drowning at home

**Fig. 4:**
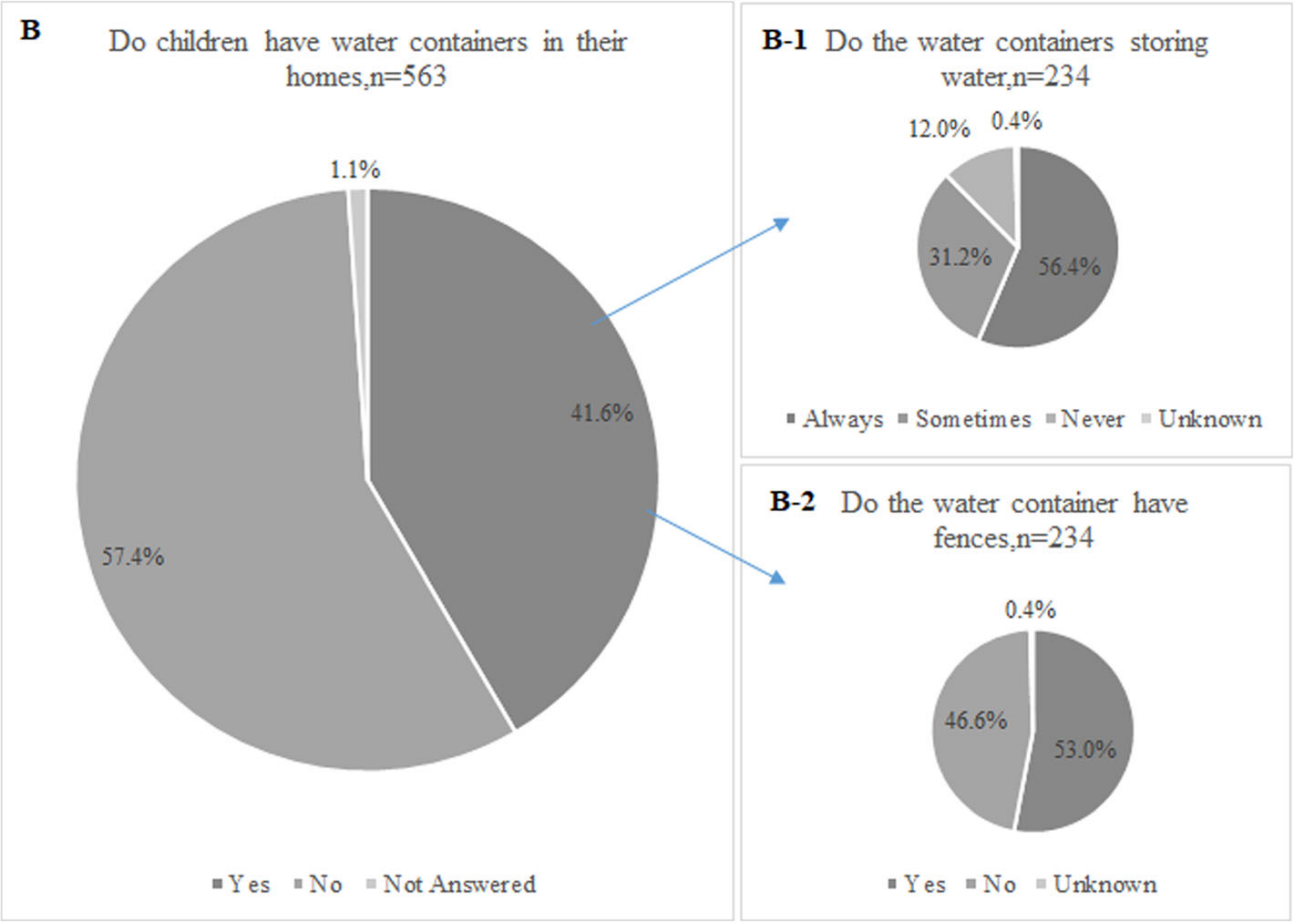
Water containers and safety measures in the houses of drowned Chinese children under 5 years old

Water containers were stored in the family homes of 234 (41.6%) drowned children (See Fig. 4). Of these containers, 56.4% always stored water, 31.2% occasionally stored water, and fewer than 12% never stored water. No safety measures, such as fences, were implemented around approximately half (46.6%) of these water containers.

## Discussion

The present study suggests the following characteristics in the drowning of Chinese children under 5 years. First, children aged 1 to 2 years old and older children who are not enrolled in kindergarten were more likely to drown. Second, most children older than 1 year old had not received any accidental injury safety education (accounted for 70.9%), and most primary caregivers (accounted for 83.1%) had not received first aid training in drowning. Third, children typically drowned in bodies of water within 100 m of their home. Younger children tended to drown at home, and older children were more likely to drown outside the home in ponds, rivers, and canals. This difference is most likely due to the mobility of older children. The children in most drowning cases were playing next to the body of water and not in the water. Fourth, safety measures were markedly lacking in most drowning cases. Finally, most drowned children had caregivers who were less educated. Caregivers were attending to other chores and not minding the child when many drownings occurred, and nearly half of all children were alone when they drowned. These findings, in combination with a previous study in Hunan Province [19], indicate that full-time caregivers, accidental injury safety training, and water safety measures are important for drowning prevention.

The present study found that a great many drowned children lived within 100 m of the body of water, and most of these bodies of water had no fences. Most public bodies of water lacked warning signs. For the public bodies of water with warning signs, half of the warning signs were not easy to notice. Since most accidental drownings occurred at ponds, canals, rivers, and wells, the incompletion of safety measures in the living environment is an important risk factor for accidental drowning in children. Therefore, physical prevention measures in parallel with drowning prevention education are needed. Our study underscores the urgent need for fences and visible warning signs around bodies of water. Local governments should be encouraged to enact regulations and policies requiring that warning signs and protective barriers be installed near public bodies of water.

One major insight from the results of this study is the role of the primary caregivers. The results suggest that primary caregivers’ education level and the level of mindfulness strongly affect the risks of child drowning. Primary caregivers in our survey were paying full attention to the child in only 12.8% of drowning cases when the primary caregiver was present. This result indicates a lapse in supervision among most caregivers.

A study in Australia showed that inadequate care and lapses in supervision, such as indoor housework, outdoor housework, and talking/socializing, were major contributors to child drowning [20]. We also found that caregivers were occupied with housework, phone calls or socializing at the time of the drowning. An Australia-based study of the accidental drowning of children from 0 to 17 years old between 2002 and 2014 found that all children who drowned had a lapse in supervision, and common supervision lapses were due to indoor household duties, outdoor household duties and talking/socializing [21]. Therefore, it is important to help primary caregivers understand the consequence of supervision lapses. Local communities can promote the benefits of full-time parental care within their jurisdictions and use health education programs to help parents learn about child drowning prevention and first aid information for drowning. Sending injury prevention knowledge via SMS or voice messages to parents’ mobile phones may effectively raise awareness and reduce the risk of drowning, as suggested by a study in Bangladesh [22]. These strategies were effective in the Jiangsu province in China, where parental awareness of, and behaviors to prevent, accidental suffocation and drowning in rural children changed significantly after the enacting of health education programs [23].

Raising awareness of drowning prevention may also prove beneficial when it is provided to the caregivers and the children. One way to increase awareness of kindergarten-aged children would be for the local government to strengthen safety education training in the classroom and encourage kindergarten enrollment because we found that over half of drowned children were not enrolled in kindergarten despite being of kindergarten age.

We propose teaching children to avoid unprotected bodies of water to help reduce the drowning mortality rate. As children become older, more mobile and independent, emphasis should be shifted from safety around water containers in the home to safety around open bodies of water outdoors. Moreover, prevention programs should reinforce that a child should always seek adult supervision when playing in and around bodies of water. To be most effective, these prevention programs should be age-appropriate.

## Conclusions

To our knowledge, the present study is the most extensive survey of the characteristics of drowning deaths of children under 5 years in China. These data suggest several key risk factors that may help guide the design and improvement of child drowning prevention programs.

## Data Availability

This study used data from the NMCHSS. This system was coestablished by the National Health and Family Planning Commission of the People Republic of China and Sichuan University, and it is owned by the National Health and Family Planning Commission of the People Republic of China. The researchers did not obtain consent to publicly share the data. The deidentified data set is available upon request to interested researchers. For data requests, please contact the Department of Science and Technology of West China Second University Hospital, Sichuan University, at: fu2yuankjb@163.com. This department is in charge of all programs in the hospital, including data management. One staff member from the department (named Xian He) monitors this email address.

## Abbreviations

AUD: Australian Dollar
NMCHSS: the National Maternal and Child Health Surveillance System
UNICEF: the United Nations Children’s Fund
SMS: Short Message Service
